# Selective hydrogenation of graphene on Ir(111): an X-ray standing wave study

**DOI:** 10.1039/d1fd00122a

**Published:** 2022-05-06

**Authors:** Claus F. P. Kastorp, David A. Duncan, Anders L. Jørgensen, Martha Scheffler, John D. Thrower, Tien-Lin Lee, Liv Hornekær, Richard Balog

**Affiliations:** Department of Physics and Astronomy, Aarhus University Aarhus Denmark balog@phys.au.dk; Diamond Light Source Ltd., Harwell Science and Innovation Campus Didcot OX11 0DE UK; The Mads Clausen Institute, SDU NanoSYD Sønderborg Denmark

## Abstract

A combined high resolution X-ray photoelectron spectroscopy and X-ray standing wave study into the adsorption structure of hydrogenated graphene on Ir(111) is presented. By exploiting the unique absorption profiles and significant modulations in signal intensity found within the X-ray standing wave results, we refine the fitting of the C 1s X-ray photoelectron spectra, allowing us to disentangle the contributions from hydrogenation of graphene in different high-symmetry regions of the moiré supercell. We clearly demonstrate that hydrogenation in the FCC regions results in the formation of a graphane-like structure, giving a standalone component that is separated from the component assigned to the similar structure in the HCP regions. The contribution from dimer structures in the ATOP regions is found to be minor or negligible. This is in contrast to the previous findings where a dimer structure was assumed to contribute significantly to the sp^3^ part of the C 1s spectra. The corrugation of the remaining pristine parts of the H-graphene is shown to increase with the H coverage, reflecting an increasing number and size of pinning centers of the graphene to the Ir(111) substrate with increasing H exposure.

## Abstract 2

Graphene on Ir(111) was hydrogenated selectively in the HCP and FCC regions by controlling the substrate temperature during exposure. Hydrogenated carbon in these areas both form ordered clusters, but are found to contribute to different components.

## Graphical abstract

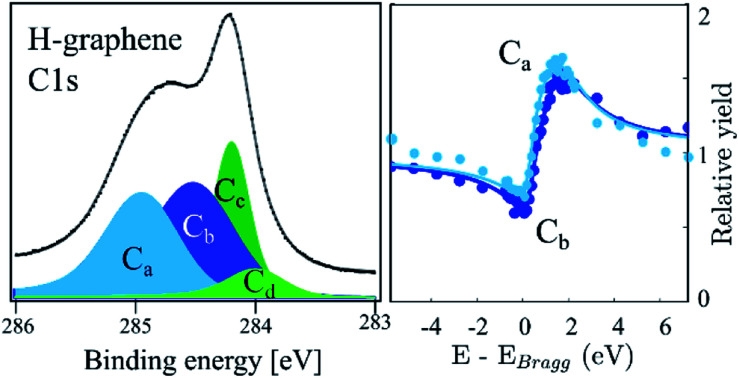

## Introduction

The chemical functionalisation of graphene with elements such as fluorine,^1,2,25^ oxygen,^8,12,13,33^ and hydrogen^3–6,18,19,23,28^ has attracted attention because of the resulting changes in material properties. For hydrogen, these effects include ferromagnetism,^23,31^ increased spin–orbit coupling,^26^ and the opening of an electronic band gap,^5,14,24,29^ which additionally can be controlled by the degree and pattern of hydrogenation.^15,19^ Other potential applications of hydrogenated graphene include protective coatings,^21^ as well as solid-state hydrogen storage.^27,30^

Graphene on Ir(111) exhibits a moiré pattern which can be closely modelled by ten-by-ten graphene unit cells for each nine-by-nine Ir surface atoms.^11^ This supercell contains three high-symmetry regions of interest, namely the HCP, FCC, and ATOP regions. In the HCP and FCC regions, every second C atom is directly above an Ir surface atom and every second above a hollow site.^11^ The C and Ir atoms interact more strongly due to the geometry, and the graphene sheet is closer to the Ir(111) surface than in the ATOP regions, 3.29 Å above the substrate for pristine graphene in the FCC regions and 3.27 Å for the HCP regions,^9^ based on *ab initio* density functional theory (DFT). In the ATOP region, where the Ir atoms are positioned directly below the centre of the carbon rings,^11^ a higher average height with a maximum of 3.62 Å is found.

It is known from earlier works that graphene on Ir(111) can be functionalised to varying levels by exposure to hot (*T* > 1700 K) atomic hydrogen, at varying substrate temperatures.^19^ We have previously shown, by a combination of scanning tunnelling microscopy (STM), X-ray standing waves (XSW) and DFT, that for hydrogenation at high substrate temperatures (HT), *ca.* 600 K, hydrogenation only occurs at the HCP regions^18,19^ leading to the formation of graphane-like clusters where the C atoms alternately bind to a hydrogen atom above the graphene and to a surface Ir atom at the graphene–Ir(111) interface.^18^ Hydrogenation at intermediate temperatures (IT), *ca.* 550 K, results in the functionalisation of both the HCP and FCC regions, as observed by scanning tunnelling microscopy (STM).^19^ It is expected that the functionalised FCC regions resemble the structures observed in HCP regions due to similar registry between the graphene sheet and the Ir surface in these two regions.

Hydrogenation at room temperature (RT), *ca.* 300 K, results in a higher level of functionalisation, as observed in STM and X-ray photoelectron spectroscopy (XPS).^4,5,19^ Since, in the ATOP region, the C and Ir atoms are out of registry, graphane-like clusters are not expected to form in the same way as in the HCP and FCC regions. As STM and DFT studies show the formation of H-dimers on both graphite^16,17^ and graphene on SiC(0001)^6^ after exposure to atomic hydrogen, it has been suggested that dimers might also form in the ATOP regions of graphene on Ir(111).^4^ The presence of dimer structures in the ATOP regions has, however, never been imaged directly by STM, as this technique itself is not capable of resolving any detailed structures at high level of functionalisation due to the large-scale changes in electronic structure induced by the hydrogenation.^5,19^

The complex evolution of the structure of hydrogenated graphene is clearly seen in the C 1s photoemission spectra, which become more complex with increasing extent of functionalisation. In earlier XPS works, the C 1s spectrum of fully saturated H-graphene exhibits a minimum of four components labelled C_a_, C_b_, C_c_, and C_d_ in order of decreasing binding energy.^4^ Here, the C_c_ component was attributed to the pristine sp^2^ carbon, and even at the saturation level of functionalisation it has a minimum of *ca.* 30% of the total C 1s intensity. The structure of this remaining pristine graphene remains unclear. Similarly, the same study reveals that the component C_b_ represents sp^3^-hybridised C in graphene-like clusters. This structure was recently determined by XSW in a sample hydrogenated at 600 K.^18^ However, the structures of the remaining two components, C_a_ and C_d_, are to date unstudied by XSW, although it has been suggested that the C_a_ component predominantly represents dimers forming in the ATOP regions and that the C_d_ component represents sp^2^-hybridised C atoms neighbouring sp^3^-hybridised C atoms.^4^

In this work, we expand the analysis of these more complicated photoemission spectra using a combination of high resolution XPS and XSW measurements obtained as a function of the substrate temperature during hydrogenation. Our results lead us to question the standard view of the assignment of peaks and provide greater insight into the selective functionalisation of graphene. In particular, in addition to presenting novel XSW results for the IT and RT hydrogenated samples, we also refine the analysis of the HT XSW data^18^ in order to fit the data consistently across the temperature dependent data sets.

## Experimental procedures

### Experimental setup

The XPS and XSW measurements were performed at the permanent end station of the I09 beamline^22^ at the Diamond Light Source. The photoelectron spectroscopy data was acquired using a Scienta EW4000 HAXPES analyser mounted in the horizontal plane at a 90° angle from the incident radiation. The light source consists of two separate undulators for providing soft and hard x-rays that irradiate onto overlapping spots on the sample. The soft X-ray light was used to obtain the high resolution XPS data (HR-XPS) and the hard X-ray light to obtain the hard X-ray photoelectron spectroscopy (HAXPES) and XSW data.

### Graphene growth and hydrogenation

Graphene was produced on the (111) face of an Ir single crystal *via* the chemical vapour deposition method.^10^

First, the room temperature sample was exposed to a pressure of *p* = 2 × 10^−7^ mbar of ethylene (C_2_H_4_) for two minutes. Then, the sample was quickly heated to 1470 K while maintaining the ethylene pressure, and then cooled to *ca.* 1220 K. At this point, the pressure of ethylene was increased to 4 × 10^−6^ mbar for four minutes. The flow of ethylene was shut off, and the sample flash annealed to 1470 K before cooling to room temperature.

Prior to the functionalisation with hot atomic hydrogen, the quality of the graphene was examined by XPS.

Hot atomic hydrogen was produced by passing molecular hydrogen gas through a tungsten capillary heated by an electric current (*T* > 1700 K). The sample was heated to the desired temperature before being exposed to the H-beam until saturation coverage was achieved for a given sample temperature (background H pressure of 5 × 10^−7^ mbar for 45 min).

Each sample was allowed to cool down to room temperature before any photoelectron measurements were performed.

### X-ray photoelectron spectroscopy and standing wave measurements

For each hydrogenated sample, several photoemission spectra of the C 1s orbital were acquired: a high-resolution spectrum acquired at a photon energy of 435 eV, as well as a measurement series of 42 HAXPES for the HT measurement and 40 HAXPES each for the IT and RT measurements with photon energies from within a narrow region around the Bragg energy. For Ir(111), this corresponds to 2798 eV at normal incidence. For each photon energy, the reflectivity of the sample was measured as well. Due to the low count rates, the XSW measurement series were repeated on several different spots on the sample, and the spectra acquired at the same photon energy were summed to obtain a single measurement series with a better signal-to-noise ratio.

XSW exploits the standing wave field created when the photon energy is close to the Bragg energy. Interference between the incoming and diffracted light creates a standing wave, whose periodicity is that of the Ir crystal between the (111)-planes, *d*^111^ = 2.216 Å. The phase variation with photon energy provides a characteristic modulation of each individual C 1s component in the spectra. This modulation depends on the average adsorption height for the component.^32^

The resulting absorption curves were fitted using dynamical diffraction theory^7^ using two dimensionless structural parameters, both taking values between 0 and 1: the coherent position (*P*^111^) and the coherent fraction (*F*^111^). The coherent position corresponds to the average height of the atomic species expressed as a fraction of the standing wavelength. A coherent position of 0 or 1 corresponds to being in-plane with the extension of the crystal lattice, and two atoms positioned exactly one lattice periodicity apart will be indistinguishable.^20,32^ Given the periodicity of the standing wavefield, the average height *h* equals *h* = *d*^111^(*n* + *P*^111^), where *n* is an integer. A value of *n* = 1 has been found to be appropriate for pristine graphene,^9^ as well as graphane-like clusters in H-graphene on Ir(111),^18^ and it will be assumed to apply throughout this article.

The coherent fraction is often interpreted as an order parameter. Flat structures will have high coherent fractions, and thus a decreasing value of the coherent fractions will often indicate an increase in the level of corrugation.^20^

In the HAXPES data, components have significantly larger Gaussian widths, which is primarily an effect of the energy resolution at different photon energies (*ca.* 0.01% of the photon energy), as well as the higher pass energy used for XSW measurements compared to HR-XPS.

### Fitting procedures

The analysis of the spectra was performed with KolXPD.^34^ All spectra have been fitted using a linear background and a number of peaks to represent individual components. Within a spectrum, all components were forced to have the same Lorentzian width. For each high-resolution spectrum, as few components as possible were used. The acquired number of components and binding energies were then used to fit the XSW spectra. All acquired fitting parameters are tabulated in Table 1.

**Table 1:** Fitting parameters for each component in the C 1s photoemission spectra, as well as for the relative absorption curves. The Lorentzian width (L. wid.) is the same for all components within a spectrum, as well as for the high-resolution spectra and the corresponding HAXPES. The HT spectra are taken at a different time and with different settings than the IT and RT spectra, and thus the difference in the Lorentzian width is of little concern. The Gaussian width (G. wid.) refers to the high-resolution spectra and is generally wider in the HAXPES. The share refers to the relative intensity of that component calculated as the intensity of each component divided by the sum of the intensities of all components in the spectrum. The spectra taken at 435 eV and 2792 eV on the same sample should provide similar shares for all components. The intensity of the C_d_ component is too low for structural analysis, and its median height is therefore omitted

	Values from ref. 18	HT	IT	RT
Err/N	3.80	2.25	1.84	1.25
L. wid./eV	0.13	0.10	0.13	0.12
C_a_ BE/eV			C_c_ + 0.73	C_c_ + 0.73
Share			10%	34%
G. wid./eV			0.60	0.62
*F* ^111^			0.61(5)	0.55(4)
*P* ^111^			0.03(2)	0.05(2)
Height/Å			2.28(4)	2.33(4)
C_b_ BE/eV	C_c_ + 0.44	C_c_ + 0.30	C_c_ + 0.30	C_c_ + 0.30
Share		33%	34%	31%
G. wid./eV		0.70	0.60	0.63
*F* ^111^	0.41(2)	0.56(6)	0.67(8)	0.65(3)
*P* ^111^	0.02(3)	0.05(3)	0.10(3)	0.02(1)
Height/Å	2.26(7)	2.33(7)	2.44(7)	2.26(2)
C_c_ BE/eV	284.18	284.18	284.24	284.19
Share		67%	56%	26%
G. wid./eV		0.26	0.24	0.27
*F* ^111^	0.48(3)	0.65(3)	0.52(2)	0.48(4)
*P* ^111^	0.56(1)	0.57(1)	0.56(1)	0.45 (2)
Height/Å	3.46(2)	3.48 (2)	3.46(2)	3.21(4)
C_d_ BE/eV				C_c_ − 0.21
Share				9%
G. wid./eV				0.46
*F* ^111^				0.21(9)
*P* ^111^				0.00(8)

Correction of the binding energies with respect to the Fermi level is possible for the HR-XPS, but it is not practical for the HAXPES due to the low count rates. Rather, the components in HAXPES and HR-XPS have been assumed to exhibit the same separations in binding energy as well as relative intensities.

When fitting the XSW spectra, all spectra acquired for one sample were described by components that can only vary in intensity across spectra, while the widths and binding energies of each component were kept constant for all photon energies. The process of finding the best possible fitting parameters is highly iterative, and several criteria have to be considered:

Firstly, any set of fitting parameters should provide as high coherent fractions as possible for all components, since higher values are mathematically more likely to represent a single atomic species.^20^

Secondly the relative intensities of the different components were assumed in the off-Bragg spectra (2792 eV) to be similar to those of the HR-XPS.

Thirdly, the Lorentzian widths, which arise due to the life-time broadening of the core hole, were set to the value obtained from the fit of the HR-XPS.

Fourthly, the C_a_ and C_b_ components in the RT spectra were forced to have the same Gaussian width in the XSW spectra, as they have similar widths in the high-resolution spectrum.

Fifthly, coherent fractions above 0.8 were considered artificially high due to the inherent corrugation within the graphene layer.

## Results and discussion

### Photoemission results

**Fig. 1:**
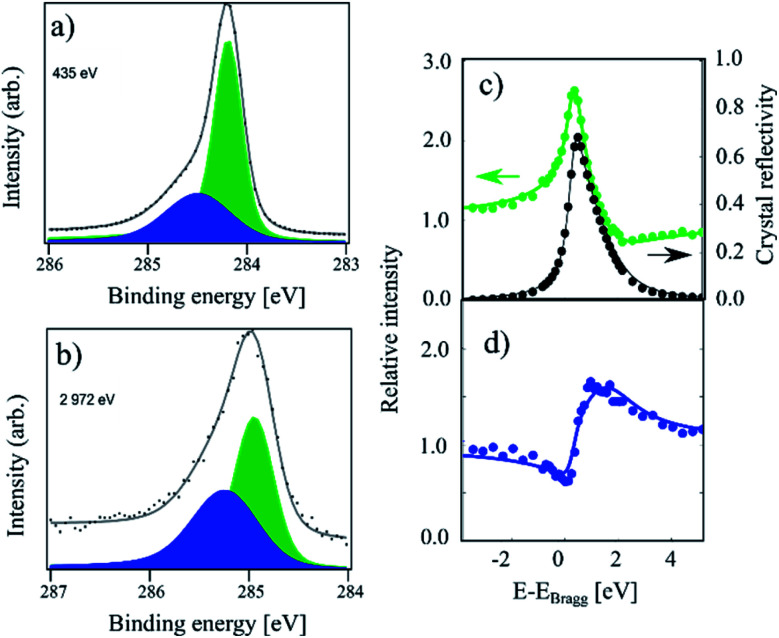
C 1s photoemission spectra acquired on a sample hydrogenated at 600 K (referred to as HT in the text). (a) The high-resolution spectrum acquired at a photon energy of 435 eV. The C_b_ component (blue) and C_c_ component (green) area separated by 0.30 eV in binding energy. (b) The same sample measured at a photon energy of 2792 eV. (c) Relative absorption of the C_c_ component with best fit to structural parameters (green) and the reflectivity of the crystal (black) as a function of photon energy. Arrows indicate proper axes. (d) Relative absorption of the C_b_ component with best fit to the structural parameters. This data has been reproduced from ref. 18 with permission from the Royal Society of Chemistry, and it has been revisited and reanalysed for this work. The exact fitting parameters can be found in Table 1.

In Fig. 1 XPS and XSW C 1s photoemission spectra acquired on a sample hydrogenated at 600 K (HT) are displayed. At this temperature, only the HCP regions are expected to functionalise.^18,19^ The HR-XPS data obtained at 435 eV photon energy and the XSW data obtained at a photon energy of 2972 eV can both be fitted using two components (C_c_ and C_b_). Previously, when analysing the HT spectra,^18^ we constrained our fits to the fitting parameters described in ref. 4. However, by using the fitting procedure laid out above and fitting the HT spectra with the two components (C_c_ and C_b_), we obtained the best fit with a binding energy shift between the two components of 0.30 eV, instead of the 0.44 eV used in the fitting of our prior work, as shown in Fig. 1. This resulted in not only a better numerical fit to the HR-XPS (normalised error of the fit decreasing from 3.80 to 2.26), but also, notably, this subtle change in the relative binding energy of the two peaks results in a significant difference in the resulting coherent fractions of both species. Specifically, for C_c_/C_b_ respectively, the coherent fractions increased from 0.41(2)/0.43(5) to 0.65(3)/0.56(7). Nevertheless, the coherent position did not change significantly, 0.56(1)/0.02(3) and 0.57(1)/0.05(3), corresponding to average heights of 3.46(2) Å/2.26(7) Å and 3.48(2) Å/2.33(7) Å, respectively. All structural values are tabulated in Table 1.

The average adsorption height for the sp^2^ species is close to the values observed for pristine graphene in other works, 3.38(4) Å in ref. 9 and 3.41(4) Å in ref. 18, although the coherent fraction is significantly higher for the measurements on pristine graphene, 0.74(4) in ref. 9 and 0.74(1) for ref. 18, indicating an increased corrugation in the HT functionalised graphene. The average adsorption height for the C_b_ component of 2.26(7) Å is in good agreement with the theoretically predicted height for C atoms in sp^3^-hybridised graphene-like clusters. As the C_b_ component comprises 33% of the overall signal, the HCP regions are likely fully hydrogenated.

**Fig. 2:**
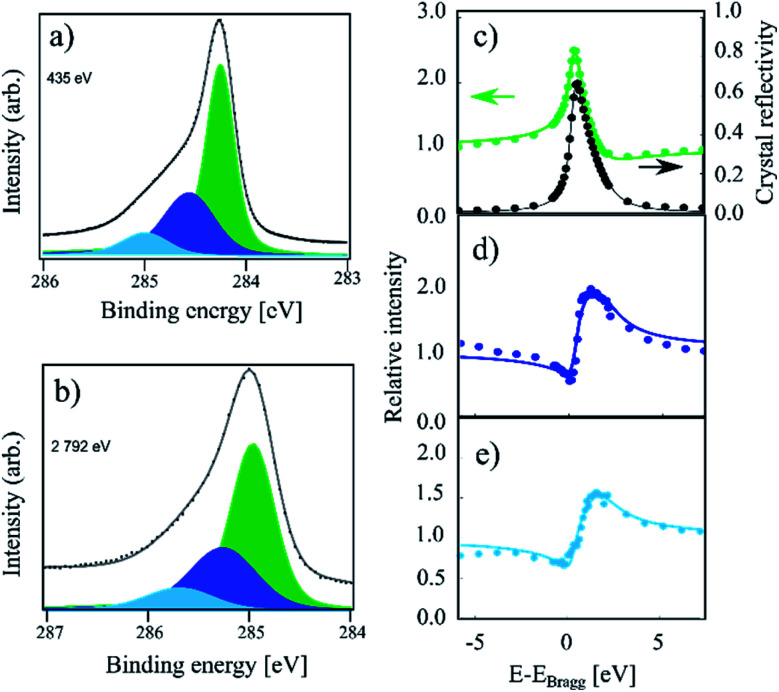
C 1s photoemission spectra acquired on a sample hydrogenated at 550 K (referred to as IT in the text). (a) The high-resolution spectrum acquired at a photon energy of 435 eV. Along with the C_b_ (dark blue) and C_c_ (green) components observed at HT, the C_a_ component (light blue) is present. (b) The same sample measured at a photon energy of 2792 eV. (c) Relative absorption of the C_c_ component with best fit to structural parameters (green) and the reflectivity of the crystal (black) as a function of photon energy. Arrows indicate proper axes. (d) Relative absorption of the C_b_ component with best fit to the structural parameters. (e) Relative absorption of the C_a_ component with best fit to the structural parameters. In the XSW spectra we see evidence of beam damage in the form of significant hydrogen desorption. This affects the quality of the XSW fits. The exact fitting parameters can be found in Table 1.

In Fig. 2 the HR-XPS and XSW measurements for the sample hydrogenated at 550 K (IT) are displayed. The intensity is visibly increased on the high binding energy end of the spectrum compared to that of HT. Utilising the described fitting approach, we fit the IT spectra with three components: C_a_, C_b_ and C_c_, as shown in Fig. 2. The binding energy for the C_a_ component was found at 0.73 eV above that of the C_c_ component, with a C_a_–C_b_ separation of 370 meV, thus 60 meV smaller than that reported in ref. 4. Fitting of the XSW data resulted in very similar coherent position for C_c_ as that found for the HT sample, 0.56(1), corresponding to 3.46(2) Å on average, though the coherent fraction decreased significantly to 0.52(2). This indicates a higher degree of corrugation in the remaining sp^2^ regions. The coherent fraction of the C_b_ component in the IT sample increased to 0.67(9) and the coherent position also increased slightly to 0.10(4)/2.44(7) Å. Both values, however, have overlapping confidence intervals with the HT values. The new C_a_ component has a coherent fraction and coherent position almost identical to those of the C_b_ component of the HT sample: 0.61(5) and 0.03(2)/2.28(4) Å, respectively.

We note that the IT spectra shows significant beam damage in the XSW spectra for the C_b_ species in the form of hydrogen desorption occurring during the measurement. This is exemplified by the poor fit of the XSW absorption profile, where the low photon energy signal is significantly more intense than the high photon energy signal. This naturally influences the quality of the fit. Our data shows that this effect is present but is far weaker in the HT and RT spectra than in the IT spectra.

**Fig. 3:**
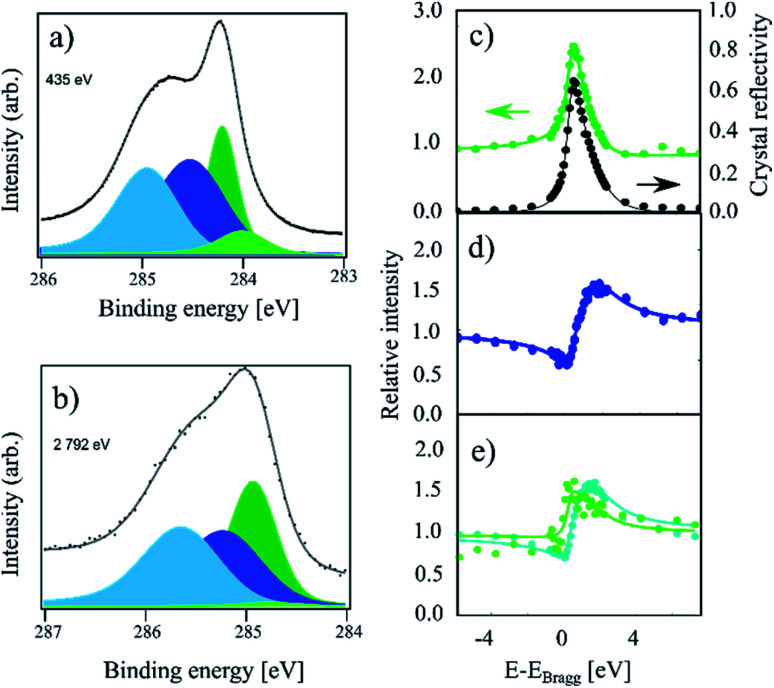
C 1s photoemission spectra of a sample which was hydrogenated at room temperature (referred to as RT in the text). (a) The high-resolution spectrum acquired at a photon energy of 435 eV. All four components, C_a_ (light blue), C_b_ (dark blue), C_c_ (dark green), and C_d_ (light green), are present. (b) Photoemission spectrum acquired at a photon energy of 2792 eV using all components, as in (a). The low intensity of the C_d_ component cannot be properly replicated at high photon energies due to the much lower resolution. (c) Relative absorption of the C_c_ component with best fit to structural parameters (green) and the reflectivity of the crystal (black) as a function of photon energy. Arrows indicate proper axes. (d) Relative absorption of the C_b_ component with best fit to the structural parameters. (e) Relative absorption of the C_a_ component (blue) and C_d_ component (green) with best fit to the structural parameters. The C_d_ component produces no discernible signal apart from a replication of the reflectivity. The exact fitting parameters can be found in Table 1.

Finally, in Fig. 3, HR-XPS and XSW spectra for the room temperature (RT) sample are presented. Compared to the IT and HT spectra, this sample exhibits a strong increase of the higher binding energy components, as well as a small shoulder at lower binding energy. To fit this spectrum, we added the fourth component C_d_ at 0.21 eV below the C_c_ component (compared to 0.27 eV from ref. 4). However, we note that C_d_ is very weak, thus the fit is likely not unique. For the RT spectrum we obtain a 34 : 31 : 26 : 9 ratio for the four components: C_a_, C_b_, C_c_ and C_d_, respectively. As the remaining C_c_ coverage is comparable to the 30% of C_c_ remaining at the highest coverage in ref. 4, we expect that this sample is hydrogenated to saturation coverage. Including a fifth component in the fit on the high binding energy side did not result in a significant improvement to the fit. The fitting of the XSW data shows a largely unchanged coherent fraction for C_c_ compared to IT at 0.48(4), but a significant decrease in the coherent position down to 0.45(2)/3.21(4) Å. C_a_ and C_b_ exhibit coherent fractions of 0.55(3) and 0.65(3), respectively, which are similar to the values obtained in the IT and HT samples, but with a slightly increased coherent position for C_a_, 0.05(4)/2.33(9) Å, as well as a slightly decreased coherent position of C_b_, 0.02(1)/2.26 Å.

Finally, the intensity of the C_d_ component is too low to be resolved in the XSW spectrum. The absorption curve for this component in Fig. 3e simply replicates the general reflectivity of the crystal with a coherent position that is indistinguishable from 0 and with a low coherent fraction. Therefore, the resulting values are most likely not representative of the structure of this species.

### Functionalised structures

We note that, in the fitting presented here, the relative coverage of the C_b_ component remains stable across all three samples (*ca.* 30% of the overall signal). As this is the case even in the HT sample, where the HCP regions are functionalized, this strongly indicates that the C_b_ component exclusively represents sp^3^-hybridised C atoms and, thus, graphene-like clusters, as observed in ref. 18 within the HCP regions. This assignment is further supported by the fact that the coherent position and the coherent fraction of the C_b_ component barely differs as a function of the sample preparation when taking the beam damage in the IT spectra into account.

The strong agreement between the values of the coherent fraction and position for the C_a_ and C_b_ components indicate that both of these two components represent the same hydrogenation structure. Therefore, both components represent graphane-like structures. In combination with previous STM and DFT results^19^ that correlate lower substrate temperatures with increasing hydrogenation in the FCC regions, this indicates that the C_a_ component relates to graphane-like structures in FCC regions.

In principle, the C_a_ component could also arise due to the increasing size of graphene-like clusters in the HCP regions when H is deposited at intermediate temperature. However, based on the deposition temperature and the high observed intensity of the sp^3^ component in the HT spectra, we expect that both the HT and IT samples contain fully functionalized HCP regions.^19^ Note that for the relative intensities of the three signals (C_a_, C_b_ and C_c_), a ratio of 10 : 34 : 56 (respectively) is observed at IT. As the 34% intensity of the overall signal in the C_b_ component is in good agreement with the value obtained for the HT sample, any additional functionalisation in the FCC regions must contribute to the C_a_ component. This is consistent with earlier STM measurements performed on both types of samples, which show that the FCC regions are functionalised to a much lower degree than the HCP regions at elevated temperatures.^19^ Therefore, hydrogenating only around a third of the FCC carbon atoms (*ca.* 10% of the overall signal) at 550 K is a realistic figure.

This significant difference in reactivity, as well as core level shift between the functionalised C atoms in the FCC and HCP regions, is striking, since the registry between the graphene sheet and the surface layer of Ir atoms is identical. Instead, the difference between the regions stems from the position of Ir atoms in the second and third Ir layer below the surface with respect to the C atom binding to H. In the more reactive HCP sites, the C atom is positioned above an Ir atoms in the third layer from the surface, while the C atoms in the less reactive FCC region are above an Ir atom in the second layer. Such an effect is surprising, but differences in the reactivity of the HCP and FCC regions of graphene in Ir(111) have previously been observed elsewhere.^5,18,19^

By comparing all three samples (HT, IT, and RT), the coherent fraction of the C_c_ component, representing the pristine parts of the graphene sheet, is seen to gradually decrease with increasing level of functionalisation, though the decrease in coherent fraction between the IT and RT cases is not significant. As the graphane-like clusters in the HCP and FCC regions grow, a larger part of the graphene sheet is pinned to the substrate. The observed lower coherent fraction is, thus, a result of an increasing corrugation in the remaining pristine parts. This is in broad agreement with previous DFT calculations.^18^ The coherent position of the C_c_ component remains constant in the HT and IT spectra compared to the value obtained for pristine graphene,^9,18^ but decreases significantly in the RT sample. This signifies that the sp^2^ C atoms are being strongly pulled down towards the Ir substrate with the increasing relative coverage of graphane-like regions from IT (41%) to RT (65–74%, the latter number including the C_d_ component). Note that most of the difference observed between the IT and RT spectra is due to increasing hydrogenation in the FCC regions rather than the ATOP regions.

What remains unclear is the extent of hydrogenation in the ATOP regions and its contribution in the RT sample to the C 1s spectra. The earlier proposed dimer structures in ATOP regions could in principle be assigned to the C_d_ component, as this component appears only in the RT spectra. It is, however, not considered likely, due to the high thermal stability of the C_d_ component.^4^

Neither is the C_a_ component likely to represent such structures. The relative area of the C_a_ component in the RT spectra is 34%, and it is only marginally more intense than the C_b_ component at 31%. As the sp^3^ C atoms in the FCC region were assigned to the C_a_ component, realistically at most 10% of the C_a_ signal (∼3% of all C atoms) may arise from hydrogenation in the ATOP regions. Our analysis reveals that there is no notable change in the structural parameters for the C_a_ component, compared to the IT sample. However, any major functionalization of ATOP regions is expected to increase the coherent position, as well as reduce the coherent fraction, due to the large spread of such H clusters in the corrugated graphene. Furthermore, there is only an insignificant decrease in the coherent fraction of the C_c_ component in going from the IT to the RT sample, indicating no significant rehybridisation. Hence, the results presented here do not support the presence of dimer structures in ATOP regions at any significant coverage.

The excess of functionalisation beyond what is expected by the hydrogenation of the FCC and HCP regions alone could be due to the H-clusters expanding to the out of registry regions at the edges of the graphane-like clusters. Such a change would be consistent with the decrease in coherent position of the C_c_ component from the IT to the RT sample, as the majority of the graphene sheet is now pinned to the substrate. Since the sp^3^ C atoms near these edges are not directly above an Ir atom, this would lead to a strong deformation around these atoms. Such regions may potentially contribute to the C_d_ component.

## Conclusions

The XPS and XSW analysis of the C 1s core level is performed for three separate samples of hydrogenated graphene on Ir(111) with varying levels of hydrogenation. At least four components are necessary for a full description of the spectra. These components are labelled C_a_, C_b_, C_c_, and C_d_ in order of decreasing binding energy. By selective hydrogenation of the moiré parts, the contributions of each part of the supercell to individual components have been identified as follows: the C_b_ component can be ascribed exclusively to sp^3^ in graphane-like carbon in the HCP regions. Hydrogenation of the FCC region results in a graphane-like configuration, as was previously determined for the hydrogenation of the HCP region.^18^ The C_a_ component is primarily related to the sp^3^ carbon in these clusters, with a possible minor contribution from ATOP region functionalisation. Our results indicate a surprising significant difference in binding energy, as well as reactivity, for the carbon atoms in the HCP and FCC regions.

The C_c_ component, representing pristine sp^2^-hybridised carbon, was shown to increase its corrugation with increasing functionalisation, and it was found to move towards the Ir substrate at the highest level of hydrogenation. Even at the saturation level of hydrogenation, this component remains at above 25% relative intensity. Finally, the C_d_ component, which only appears in samples hydrogenated at room temperature, has too low an intensity for a confident analysis using XSW.

## Conflicts of interest

There are no conflicts of interest to declare.

## Supplementary Material

## References

[cit1] Akhtar N., Anemone G., Farias D., Holst B. (2019). Fluorinated graphene provides long lasting ice inhibition in high humidity. Carbon.

[cit2] Avsar A., Lee J. H., Koon G. K. W., Özyilmaz B. (2015). Enhanced spin–orbit coupling in dilute fluorinated graphene. 2D Mater..

[cit3] Balakrishnan J., Koon G. K. W., Jaiswal M., Castro Neto A. H., Özyilmaz B. (2013). Colossal enhancement of spin–orbit coupling in weakly hydrogenated graphene. Nat. Phys..

[cit4] Balog R., Andersen M., Jørgensen B., Sljivancanin Z., Hammer B., Baraldi A., Larciprete R., Hofmann P., Hornekaer L., Lizzit S. (2013). Controlling hydrogenation of graphene on Ir (111). ACS Nano.

[cit5] Balog R., Jørgensen B., Nilsson L., Andersen M., Rienks E., Bianchi M., Fanetti M. (2010). *et al.*, Bandgap opening in graphene induced by patterned hydrogen adsorption. Nat. Mater..

[cit6] Balog R., Jørgensen B., Wells J., Lægsgaard E., Hofmann P., Besenbacher F., Hornekær L. (2009). Atomic hydrogen adsorbate structures on graphene. J. Am. Chem. Soc..

[cit7] Batterman B. W. (1964). Effect of dynamical diffraction in X-ray fluorescence scattering. Phys. Rev..

[cit8] Buchsteiner A., Lerf A., Pieper J. (2006). Water dynamics in graphite oxide investigated with neutron scattering. J. Phys. Chem. B.

[cit9] Busse C., Lazić P., Djemour R., Coraux J., Gerber T., Atodiresei N., Caciuc V. (2011). *et al.*, Graphene on Ir (111): physisorption with chemical modulation. Phys. Rev. Lett..

[cit10] Coraux J., Engler M., Busse C., Wall D., Buckanie N., Meyer Zu Heringdorf F.-J., Van Gastel R., Bene P., Michely T. (2009). Growth of graphene on Ir (111). New J. Phys..

[cit11] N’Diaye A. T., Coraux J., Plasa T. N., Busse C., Michely T. (2008). Structure of epitaxial graphene on Ir (111). New J. Phys..

[cit12] Dikin D. A., Stankovich S., Zimney E. J., Piner R. D., Dommett G. H., Evmenenko G., Nguyen S. T., Ruoff R. S. (2007). Preparation and characterization of graphene oxide paper. Nature.

[cit13] Dreyer D. R., Park S., Bielawski C. W., Ruoff R. S. (2010). The chemistry of graphene oxide. Chem. Soc. Rev..

[cit14] Elias D. C., Nair R. R., Mohiuddin T. M. G., Morozov S. V., Blake P., Halsall M. P., Ferrari A. C., Boukhvalov D. W., Katsnelson M. I., Geim A. K., Novoselov K. S. (2009). Control of graphene’s properties by reversible hydrogenation: evidence for graphane. Science.

[cit15] Haberer D. V. V. D., Vyalikh D. V., Taioli S., Dora B., Farjam M., Fink J., Marchenko D., Pichler T., Ziegler K., Simonucci S., Dresselhaus M. S. (2010). Tunable band gap
in hydrogenatedquasi-free-standing graphene. Nano Lett..

[cit16] Hornekær L., Rauls E., Xu W., Šljivančanin Ž., Otero R., Stensgaard I., Lægsgaard E., Hammer B., Besenbacher F. (2006). Clustering of chemisorbed H (D) atoms on the graphite (0001) surface due to preferential sticking. Phys. Rev. Lett..

[cit17] Hornekær L., Šljivančanin Ž., Xu W., Otero R., Rauls E., Stensgaard I., Lægsgaard E., Hammer B., Besenbacher F. (2006). Metastable structures and recombination pathways for atomic hydrogen on the graphite (0001) surface. Phys. Rev. Lett..

[cit18] Jørgensen A. L., Duncan D. A., Kastorp C. F. P., Kyhl L., Tang Z., Bruix A., Andersen M. (2019). *et al.*, Chemically-resolved determination of hydrogenated graphene–substrate interaction. Phys. Chem. Chem. Phys..

[cit19] Jørgensen J. H., Cabo A. G., Balog R., Kyhl L., Groves M. N., Cassidy A. M., Bruix A. (2016). *et al.*, Symmetry-driven band gap engineering in hydrogen functionalized graphene. ACS Nano.

[cit20] The X-Ray Standing Wave Technique: Principles and Applications, ed. A. Kazimirov, World Scientific, Hackensack, NJ, 2013

[cit21] Kyhl L., Balog R., Cassidy A., Jørgensen J., Grubisic-Čabo A., Trotochaud L., Bluhm H., Hornekær L. (2018). Enhancing graphene protective coatings by hydrogen-induced chemical bond formation. ACS Appl. Nano Mater..

[cit22] Lee T.-L., Duncan D. A. (2018). A two-color beamline for electron spectroscopies at diamond light source. Synchrotron Radiat. News.

[cit23] Lee W. -K., Whitener Jr K. E., Robinson J. T., Sheehan P. E. (2015). Patterning Magnetic Regions in Hydrogenated Graphene *Via* E-Beam Irradiation. Adv. Mater..

[cit24] McKay H., Wales D. J., Jenkins S. J., Verges J. A., de Andres P. L. (2010). Hydrogen on graphene under stress: molecular dissociation and gap opening. Phys. Rev. B: Condens. Matter Mater. Phys..

[cit25] Nair R. R., Ren W., Jalil R., Riaz I., Kravets V. G., Britnell L., Blake P., Schedin F., Mayorov A. S., Yuan S., Katsnelson M. I. (2010). Fluorographene: a two-dimensional counterpart of Teflon. Small.

[cit26] Neto A. H. C., Guinea F. (2009). Impurity-induced spin–orbit coupling in graphene. Phys. Rev. Lett..

[cit27] Schlapbach L. , and Züttel A., Hydrogen-storage materials for mobile applications, Materials for Sustainable Energy: a Collection of Peer-Reviewed Research and Review Articles from Nature Publishing Group, 2011, pp. 265–270

[cit28] Smith D., Howie R. T., Crowe I. F., Simionescu C. L., Muryn C., Vishnyakov V., Novoselov K. S., Kim Y. J., Halsall M. P., Gregoryanz E., Proctor J. E. (2015). Hydrogenation of graphene by reaction at high pressure and high temperature. ACS Nano.

[cit29] Sofo J. O., Chaudhari A. S., Barber G. D. (2007). Graphane: A two-dimensional hydrocarbon. Phys. Rev. B: Condens. Matter Mater. Phys..

[cit30] Tozzini V., Pellegrini V. (2013). Prospects for hydrogen storage in graphene. Phys. Chem. Chem. Phys..

[cit31] Ulybyshev M. V., Katsnelson M. I. (2015). Magnetism and interaction-induced gap opening in graphene with vacancies or hydrogen adatoms: Quantum Monte Carlo study. Phys. Rev. Lett..

[cit32] Woodruff D. P. (2007). Adsorbate structure determination using photoelectron diffraction: Methods and applications. Surf. Sci. Rep..

[cit33] Xu Z., Sun H., Zhao X., Gao C. (2013). Ultrastrong fibers assembled from giant graphene oxide sheets. Adv. Mater..

[cit34] KolXPD, Version 1.8.0 (Build 65); Software for Spectroscopy Data Measurement and Processing, https://www.kolibrik.net/kolxpd/, accessed Dec. 2021

